# Mechanical and Electrochemical Performance of Carbon Fiber Reinforced Polymer in Oxygen Evolution Environment

**DOI:** 10.3390/polym8110393

**Published:** 2016-11-08

**Authors:** Ji-Hua Zhu, Liangliang Wei, Guanping Guo, Aizhu Zhu

**Affiliations:** 1 Guangdong Province Key Laboratory of Durability for Marine Civil Engineering, School of Civil Engineering, Shenzhen University, Shenzhen 518060, Guangdong, China; weiliangliang@email.szu.edu.cn (L.W.); guoguanping@email.szu.edu.cn (G.G.); 2 School of Civil Engineering and Mechanics, Huazhong University of Science and Technology, Wuhan 430074, Hubei, China; zhuaizhu1228@hust.edu.cn

**Keywords:** polarization, oxygen evolution, CFRP, impressed current cathodic protection, tensile strength, failure modes

## Abstract

Carbon fiber-reinforced polymer (CFRP) is recognized as a promising anode material to prevent steel corrosion in reinforced concrete. However, the electrochemical performance of CFRP itself is unclear. This paper focuses on the understanding of electrochemical and mechanical properties of CFRP in an oxygen evolution environment by conducting accelerated polarization tests. Different amounts of current density were applied in polarization tests with various test durations, and feeding voltage and potential were measured. Afterwards, tensile tests were carried out to investigate the failure modes for the post-polarization CFRP specimens. Results show that CFRP specimens had two typical tensile-failure modes and had a stable anodic performance in an oxygen evolution environment. As such, CFRP can be potentially used as an anode material for impressed current cathodic protection (ICCP) of reinforced concrete structures, besides the fact that CFRP can strengthen the structural properties of reinforced concrete.

## 1. Introduction

The durability of reinforced concrete structures can be significantly deteriorated due to the impact of rebar corrosion [1]. Different methods have been developed to mitigate steel corrosion in concrete, including the using of chemical inhibitors, surface coatings and non-corrosive steels. Research has shown that the impressed current cathodic protection (ICCP) technique effectively prevents the deterioration of reinforced concrete by preventing steel corrosion. The ICCP technique has therefore become the most promising technique in preventing the deterioration of reinforced concrete [2]. Extensive studies relating to ICCP techniques can be found in recent publications [2,3].

To effectively apply an ICCP technique in practice, it is critical to choose an anode material with a high-performance capacity in a concrete system, especially for high-resistivity reinforced concrete [4]. Current research on anode materials, optimizing for polarization behavior and cost, generally focuses on thermally sprayed zinc [5], titanium anodes [6,7], titanium mesh anodes [8], coating-overlay anodes [9], and conductive paints [10]. However, they have several drawbacks and are not well adopted in fields. For example, the incompatibility between concrete material and the current anode systems induces the vulnerability of the reinforced concrete to a harsh environment in the long term. It is therefore of great interest to develop new anode materials to prevent corrosion in reinforced concrete.

Carbon fiber-reinforced polymer (CFRP) is composed of a polymer matrix assembled with relatively strong and light carbon fibers. Due to its relatively high strength performance, CFRP has been widely used to improve the mechanical properties of reinforced concrete structures by firmly adhering CFRP to the exterior surface of concrete [11,12,13,14,15,16,17,18,19], and has been recognized a suitable material to enhance the mechanical properties of reinforced concrete [20]. However, CFRP is of relatively high conductivity and its polarization potential is comparable to that of noble metals, which may produce a galvanic corrosion problem in the CFRP reinforced concrete [21]. Hence, it is of great interest to investigate the potential of CFRP as an anode material used in a system with ICCP techniques. If CFRP has a superior performance as an anode material, together with its strong mechanical properties, CFRP would play a significant role in producing a high-performance concrete with high strength and excellent durability. These two perspectives of CFRP in reinforced concrete have been studied in several publications [4,22,23]. The experimental study from Lee-Orantes, et al. [22] presented the use of CFRP as an anode in an ICCP system to protect reinforced concrete prisms. The testing results from Nguyen, et al. [23] showed the electrochemical performance of CFRP fabric and rods either in a calcium solution or concrete. In the study from Lambert, et al. [4], CFRP was applied in pre-corroded reinforced concrete beams to understand the ICCP system and mechanical performance of CFRP. Results showed a minor decrease of the ultimate tensile strength of CFRP specimens for dual functions (strength enhancing and ICCP property) compared to the control specimens, where CFRP was only used to strengthen the mechanical properties.

To summarize, the current research has focused on the mechanical properties and corrosion behaviors of reinforced concrete with CFRP as an anode in ICCP systems. However, few studies have been performed to understand electrochemical performance of CFRP itself. Recently, it was reported that the mechanical properties of CFRP reinforced concrete stabilized when a CFRP plate was served as an anode in an ICCP system [24]. Further investigations on the failure mechanisms have been reported in [25,26]. A more systematic research is then presented in this paper, regarding the tensile strength, electrochemical performance, and tensile-failure modes of CFRP after polarization in an oxygen evolution environment. An ICCP system was simulated to study the electrochemical performances of CFRP in a NaOH solution during anodic polarization. The results include tensile-failure modes and mechanical strengths of the CFRP specimens. In addition, the impact of impressed charge density on the tensile strengths was presented and discussed.

## 2. Experimental Investigation

### 2.1. Material and Specimens

**Figure 1:**
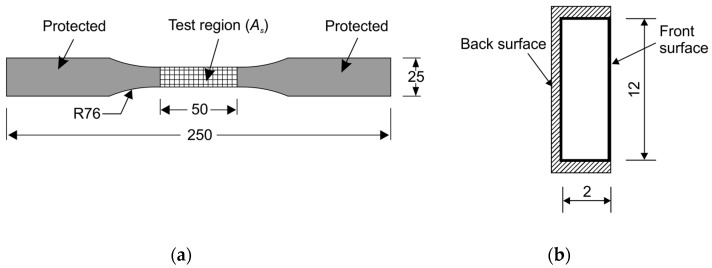
Dimensions of a carbon fiber-reinforced polymer (CFRP) specimen (mm). (**a**) front view; and (**b**) cross-sectional view.

CFRP strips were supplied by CA.BEN Composite Co., Ltd. (Hong Kong, China) and their components are multi-layer carbon fibers (Toray T700, supplied by the CA.BEN Composite Co., Ltd., Hong Kong, China) with a volume fraction of 60% mixed in LAM-125/LAM-226 epoxy (The Pro-Set Inc., Bay City, MI, USA). Table 1 presents the ingredients and their concentration of the epoxy used in the CFRP. The materials were also used in [25,26]. CFRP specimens were cut to ones with a dumbbell shape (Figure 1a) for mechanical testing in accordance with American Society for Testing and Materials (ASTM) Standard D638-10 [27]. Kafuter K-5704RTV sealant (Guangdong Hengda New Materials Technology Co., Ltd., Huizhou, China) was applied to the exterior surfaces (except the front surface in the center of the specimen, as shown in Figure 1b) to protect the specimens. The region in the center of the specimen has the nominal anodic surface area of 650 mm^2^, which is subjected to the anodic polarization in the test. Figure 1 shows the test region, protected region and the dimensions of the testing specimens.

### 2.2. Methodology

#### 2.2.1. Accelerated Polarization Test

**Figure 2:**
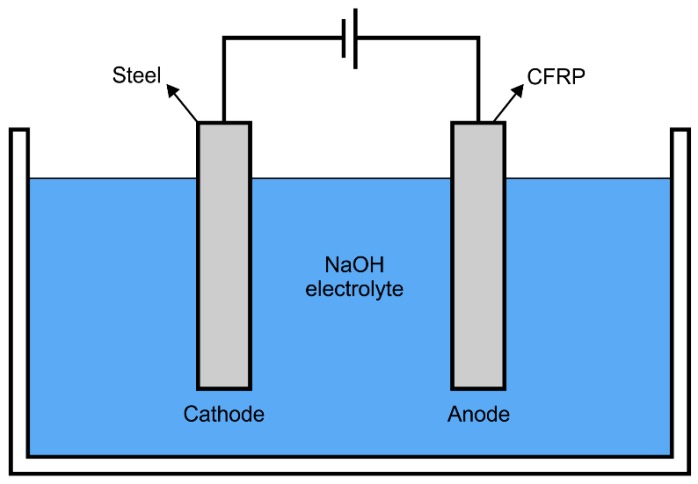
Illustrations of the impressed current cathodic protection (ICCP) system in the test.

Figure 2 illustrates the simulated ICCP system which includes an impressed current anode of CFRP, a power source, an aqueous NaOH electrolyte solution, a cathode (stainless steel strip) and a saturated calomel electrode (SCE). This ICCP system was designed to conduct the accelerated polarization tests where the galvanostatic anodic polarization behavior of CFRP was examined. By connecting the positive terminal of the power source to the specimen, the anodic polarization of CFRP was achieved. The exposed surface area of the cathode (stainless steel) equaled the test area of CFRP (*A*_s_). Hence, a generally uniform electric field distribution was achieved between the cathode and anode.

Galvanostatic anodic polarization was achieved by supplying a constant current. Different nominal current densities of 0, 0.77, 1.54, 3.08, and 6.15 A/m^2^ were controlled by applying different currents of 0, 0.5, 1, 2, and 4 mA, respectively. The specimens tested with the current value of 0 mA were selected as reference specimens. For each constant current, testing durations were set to 25 and 50 days to achieve different anodic polarization extent. Therefore, ten tests were examined in this work. 

Table 2 lists the specimens with different applied currents and polarization durations. For instance, the specimen with the ID of I2D50# was subjected to the nominal current I of 2 A for an anodic polarization duration D of 50 days. Results were obtained from the average from two replicate specimens, of which the second specimen was labeled with #. In addition, Table 2 presents key parameters relevant in the post-polarization tensile tests.

Feeding voltage between the CFRP anode and stainless steel cathode as well as the potential of CFRP versus SCE were measured every 10 min to assess the anodic properties of CFRP in the entire test period. As the feeding voltage is produced by the applied current and the electrical resistance, the measurement of feeding voltage with elapsed test time at the constant current, is adopted to demonstrate the variation of resistance in the circuit between the anode and cathode. Hence, the stability of CFRP as an anode material can be illustrated. Since feeding voltage is closely related to potential, the potential of CFRP versus SCE is also measured to examine the performance of the CFRP anode.

#### 2.2.2. Tensile Test

Post-polarization CFRP specimens were used for uniaxial tensile tests conducted on a universal test machine (E45, MTS, Eden Prairie, MN, USA) with a constant loading rate value of 0.2 mm/min. By using a data measurement system, the load applied on the specimen and the specimen displacement produced were simultaneously logged. Since the rough surfaces of a heavily corroded specimen can cause inaccuracy in the strain and cross-sectional area value, the strain value was not recorded and the cross-sectional area was measured prior to the polarization process. Tensile strength was obtained by dividing the ultimate loads by the cross-sectional area in the test region prior to polarization treatment.

#### 2.2.3. Microstructural Observations

The microstructure of CFRP specimen was imaged by using a scanning electron microscope (SEM) with the secondary electron (SE) mode. The accelerating voltage and working distance were set to 15 kV and around 10 mm, respectively. Sputter-coating was carried out to create a conductive layer of gold metal on the specimen surface. Subsequently, the specimen was loaded into the SEM chamber.

## 3. Results and Discussion

### 3.1. Anode Performance

**Figure 3:**
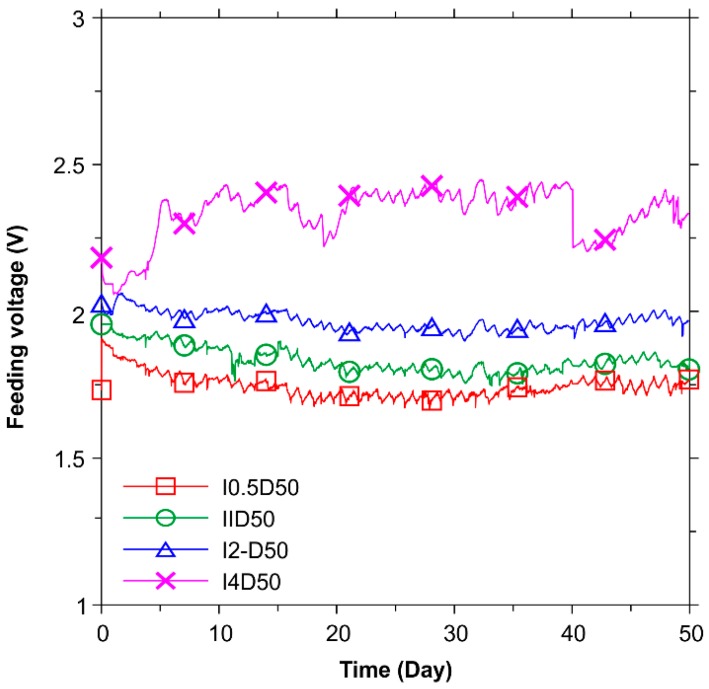
Results of feeding voltage with elapsed test time in the galvanostatic anodic polarization tests.

Figure 3 presents the results of the feeding voltages for the test duration of 50 days. For currents between 0.5, 1, 2, and 4 mA, the feeding voltage values fluctuated and stabilized at between 1.7 and 2.4 V. This indicates that CFRP can serve as the impressed current anode in a system with the ICCP technique considering its good stability.

**Figure 4:**
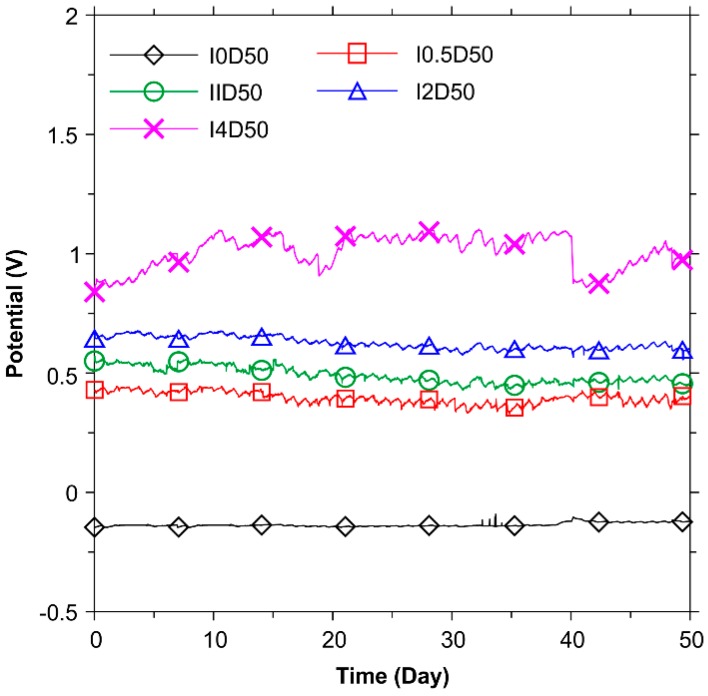
Results of CFRP potential (vs. saturated calomel electrode (SCE)) in the galvanostatic anodic polarization tests.

Figure 4 presents the results of potentials for the test duration of 50 days. For currents between 0.5, 1, 2 and 4 mA, the potential values fluctuated and stabilized at between 0.3 and 1.1 V. For the reference specimen, the potential value was found to be around −0.136 V. As expected, the potential presents a similar variation trend with feeding voltages, indicating that CFRP can have a stable anode performance in NaOH solution.

The values of feeding voltage and potential for specimen I4D50 dropped sharply at the elapsed time of 40 days. This was resulted from a power interruption in the lab. After the recovery of power supply, the measurement of feeding voltage and potential recovered.

### 3.2. Mechanical Strength and Tensile-Failure Modes

Table 2 shows the tensile strengths of post-polarization CFRP specimens and the influence of current density on the tensile strength. Results show that tensile strength decreased with current density and polarization duration. For example, the average tensile strength for the reference specimens (I0D25, I0D25#, I0D50, and I0D50#) is 682.83 MPa, and the average tensile strength for polarization specimens I4D50 and I4D50# (polarization duration is 50 days and current density is 6.392 A/m^2^) significantly dropped to 115.17 MPa. Therefore, CFRP materials were sensitively deteriorated by polarization treatment.

**Figure 5:**
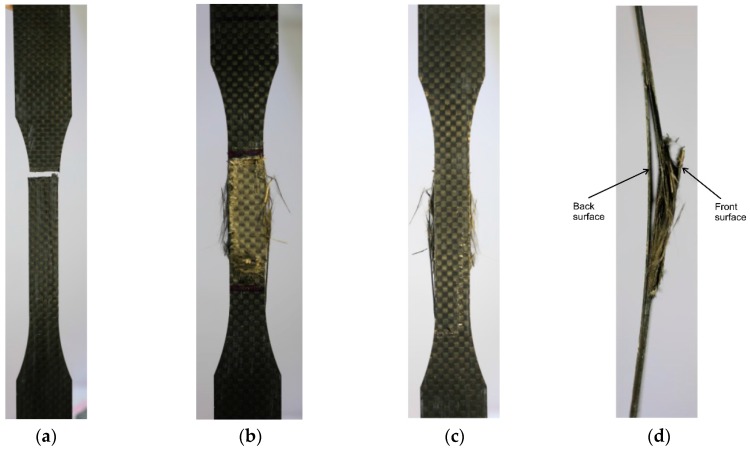
Results of two tensile-failure modes for CFRP specimens. (**a**) L (lateral) failure mode; (**b**) D (edge delamination) failure mode—front surface; (**c**) D failure mode—back surface; (**d**) D failure modes—side view.

Table 2 and Figure 5 show two distinct tensile-failure modes. Figure 5a shows the first failure mode which presents a lateral failure pattern within the gauge lengths of the specimens, and thus is named “lateral (L) mode” in accordance with ASTM Standard D3039/D3039M [28]. This L mode was also reported by Hara, et al. [29] and recognized as the typical tensile-failure mode for CFRP material. The L mode occurred in specimens that were subjected to lower current density and shorter polarization duration.

Figure 5b–d show the second failure mode which presents a vertical failure pattern across the specimen lengths with edge delamination, and thus is named “edge delamination (D) mode” in accordance with ASTM Standard D3039/D3039M [28]. The D mode occurred in specimens that were subjected to higher current density and longer polarization duration. As shown in Figure 5b,c, the front surface of a CFRP specimen has a different extent of polarization compared to the back surface because the front surface was exposed to the NaOH solution while the back surface was sealed by a sealant. This can also be observed through the side view in Figure 5d, where the right side (front surface in Figure 5b) shows more severe damage (severe corrosion destroyed the epoxy matrix) compared to the left side (back surface in Figure 5b). Hence, the D failure mode is induced by the oxidation of the epoxy polymer at the anode region.

**Figure 6:**
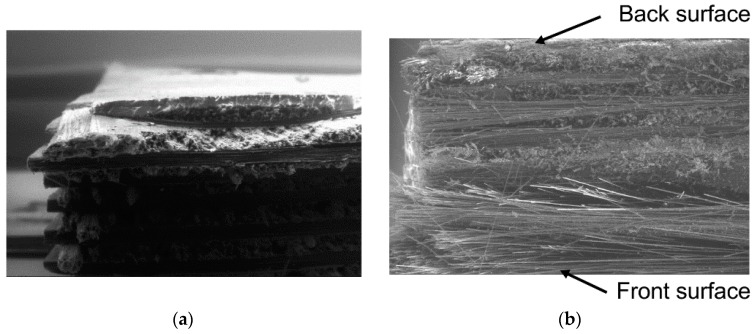
Scanning electron microscope (SEM) images of CFRP samples. (**a**) as-received; and (**b**) polarized sample with D failure mode.

The microscopic morphology of the as-received and polarized samples in D failure mode was investigated and understood by SEM images, as shown in Figure 6. The micrograph shows that the polarized sample with D failure mode has clear morphology indicating the decomposition of CFRP’s epoxy polymer at the anode test region, with only carbon fibers remaining. Sloan, et al. [30] studied the galvanically induced degradation of a graphite/epoxy cathode system, discovering three competing reactions in an alkaline environment. The reduction of oxygen was accompanied by the oxidization of epoxy polymers in CFRP. The related degradation mechanism was also demonstrated in the authors’ previous research [26], in which the epoxy in CFRP was subjected to anodic polarization and subsequently corroded, causing a variation to be noted in the CFRP’s mechanical properties.

### 3.3. Correlation between Tensile Strength and Applied Charge Density

**Figure 7:**
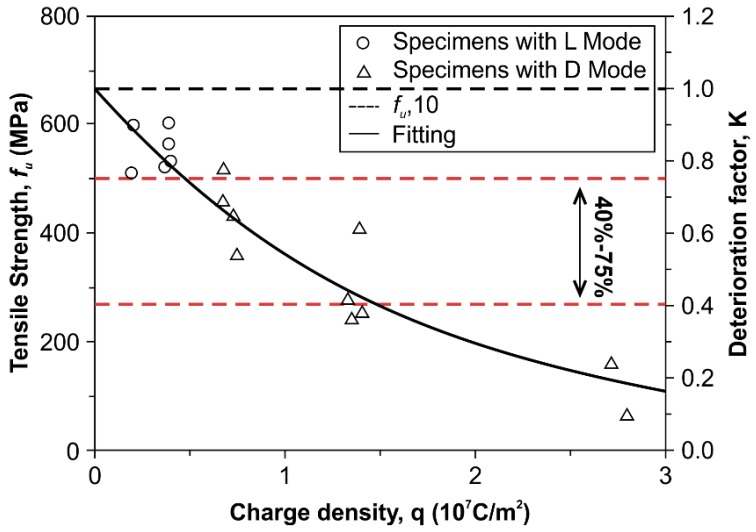
Influence of charge density on tensile strength and deterioration factor for CFRP specimens with anodic polarization in NaOH solution.

Figure 7 presents the correlation between tensile strength and charge density (*q*) applied on CFRP specimens. Note that *q* is calculated by dividing the total charge by the area of the test region and multiplying a specific time t. *A*_s_ shown in Section 3.2, tensile strength of CFRP decreases with charge density. Therefore, Equation (1) was then suggested to describe the correlation between tensile strength and applied charge density:

*f*_u_ = *Kf*_u,I0_ = *g*(*q*)*f*_u,I0_, (1)

where *f*_u_ is the tensile strength of CFRP material; *f*_u,I0_ is the average tensile strength of CFRP specimens I0D25 and I0D50 (is 682.83 MPa in this work); *K* is defined as the deterioration factor of tensile strength, and is a function of *q* (unit: 10^7^ C/m^2^), as shown in Equation (2):

*K* = *g*(*q*) = e^−0.609*q*^, (2)

According to Equations (1) and (2), the tensile strength can be obtained by multiplying a constant value (682.83 MPa in this work) with charge density. Table 2 lists the deterioration factors derived from experimental (*K*_Exp_) compared to that derived from calculated (*K*_Cal_) according to Equation (2) for CFRP specimens. Results show that the experimental value is close to the calculated, and their coefficient of variation (COV) is 0.239. Therefore, the proposed model can effectively predict the tensile strength of CFRP specimens.

### 3.4. Service Life Discussion

This paper presents the study on the mechanical and electrochemical properties of CFRP specimens to examine the feasibility of CFRP as an anode material in an ICCP system and a strength enhancing material and thus to improve the mechanical properties and the durability of reinforced concrete structures. The service life of CFRP itself is therefore critical and discussed hereafter, as suggested in [24].

Characterized by the capacity of charge transferring through the anode/electrolyte interface, the service life of a current anode can be assessed by the NACE (National Association of Corrosion Engineers) specification [31]. The capacity of charge transferring is defined as the total charge quantity (*Q*_anode_) passed by the anode in an ICCP system, and can be obtained by Equation (3):

*Q*_anode_*=i*_a_*×t*_g_*×A*_a_, (3)

where *i*_a_ is anodic current density, *t*_g_ is the ICCP duration, and *A*_a_ is the area of anodic surface.

According to the testing results, the tensile strength of CFRP decreases with charge density, as can be seen in Figure 7. In general, CFRP contributes 40% to 75% of the strength of CFRP reinforced concrete [32,33,34,35]. In order to maximize the efficiency of CFRP in a dual-function system within concrete and reach the requirements for mechanical strengthening, the residual strength of CFRP after polarization should exceed a threshold value. Figure 7 shows that a charge density value smaller than 1.372 × 10^7^ C/m^2^ can induce a tensile strength of polarized CFRP specimens satisfying the 40% tensile strength limit, and thus the threshold value of 1.372 × 10^7^ C/m^2^ can make the tensile strengths of CFRP specimens meet the requirement for mechanical strengthening. This requirement can be adjusted to measure the service life of the dual-functioning CFRP, which acts both as an impressed anode and structural strengthening material. Equation (4) describes the capacity to transfer charge (*Q*_CFRP_) of the CFRP anode:

*Q*_CFRP_ = *Q*_anode_*= i*_a_*× t*_g_*× A*_a_ = 1.372 × 10^7^*A*_a_ (C), (4)

It is known that the service life of an ICCP system is influenced by *Q*_anode_, the configuration of steel reinforcement, as well as other factors including the concrete and anode/concrete interfacial properties. However, this work concentrates on the properties of CFRP. To study the effect of *Q*_anode_ on service life, it is reasonable to assume that *Q*_anode_ is the main factor. Hence, the service life of an ICCP system can be estimated by looking at the charge quantity equilibrium between the cathode (*Q*_cathode_) and anode (*Q*_anode_), as presented in Equation (5):

*Q*_cathode_ = *Q*_anode_, (5)

**Figure 8:**
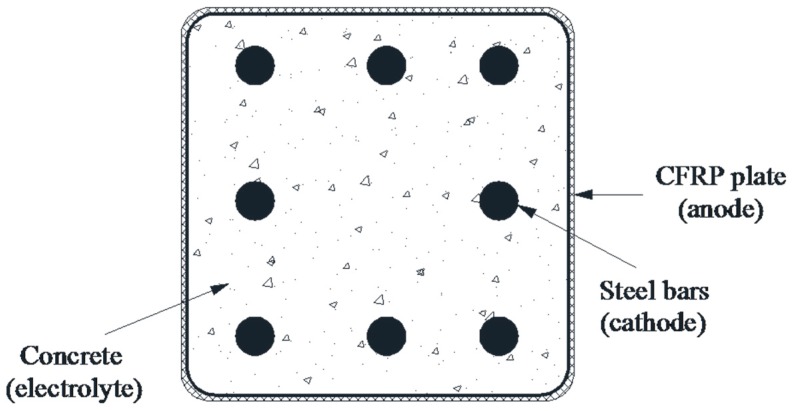
A typical reinforced concrete section with eight steel rebars and an exterior CFRP plate wrapped as an anode.

A typical concrete cross-section was used to study the service life, as shown in Figure 8. Eight steel rebars with an equal length value were used to reinforce the concrete element with a cross-section of 400 × 400 mm^2^, and acted as the cathode. A CFRP plate was wrapped on the exterior of concrete element and acted as anode. It was assumed that each steel rebar received the same current density (*i*_p_), and Equations (3)–(5) were used.

Considering a unite length of a concrete element, the charge quantity of steel cathode (*Q*_steel_) could be obtained in Equation (6):

*Q*_cathode_ = *Q*_anode_ = *n × A*_steel_*× i*_p_*× t*_life_ = *i*_p_*× t*_life_*×* (4π*nA*_c_*ρ*)^0.5^, (6)

where *n* is the number of steel rebars; *A*_steel_ is the surface area of steel per unit length contacting with the concrete; *i*_p_ is the applied current density of the cathode; *A*_c_ is the cross-sectional area of the concrete element; *ρ* is the reinforcement ratio of the concrete element, obtained by dividing the cross-sectional area of concrete by the total cross-sectional area of the steel rebar; and *t*_life_ is the service life of the ICCP system governed by *Q*_CFRP_.

Therefore, it is possible to calculate *t*_life_ by inserting Equation (6) into Equation (5). As recommended by Wyatt [36], current density of 2–20 mA/m^2^ is used for an ICCP system in reinforced concrete structures subjected to corrosion deterioration. The calculation applied current densities with an upper limit of 20 mA/m^2^ for the ICCP system, and reinforcement ratios between 0.6% and 5%, which are often adopted for designing concrete structures.

**Figure 9:**
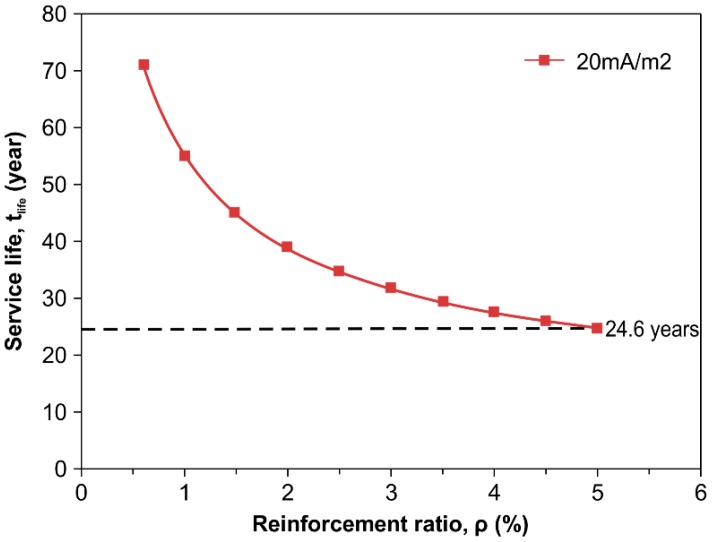
Comparison of predicted service life against reinforced ratio in an ICCP system for reinforced concrete structures.

The *t*_life_ as a function of *ρ* is shown in Figure 9. *t*_life_ decreases as *ρ* increases. With a protection current density of 20 mA/m^2^, service lives of 71 and 24.6 years are achieved, with equivalent reinforcement ratios of 0.6% and 5%, respectively. This shows that it is possible to successfully use the CFRP plate as an anode material in the ICCP system for an acceptable service period, and the mechanical properties do not degrade substantially.

Clearly, this is a relatively conservative prediction for the service life, given that the polarization was carried out in a simulated ICCP system using a much more severe corrosion environment than that of practical concrete structures.

## 4. Conclusions

This paper presents an experimental work comprehensively to examine the electrochemical and mechanical properties of CFRP in an oxygen evolution environment. Ten accelerated polarization tests were undertaken in a simulated ICCP system with NaOH solution. The main conclusions arising from this study are as follows:

1. CFRP can be potentially used as an anode material which has a stable function in an ICCP system with an oxygen evolution environment. This was indicated by the stable feeding voltage and potential measured during the polarization process.
2. The applied charge density significantly influenced the tensile strength of CFRP, which decreased with the charge density. Two typical tensile-failure modes, L (lateral) modes and D (edge delamination) modes, occurred during the polarization process. As the impressed current density and test durations increased, the failure mode changed from L mode to D mode.
3. Using the experimental results, a theoretical model was calibrated and developed to predict the tensile strength of CFRP based on specific charge densities. The calculated tensile strengths fitted the experimental data well.
4. It was shown that CFRP plates could serve well to strengthen the mechanical property as well as to protect corrosion as anode materials in reinforced concrete structures. Even with the maximum acceptable current density and reinforcement ratios, the minimum service life was conservatively predicted to be 24.6 years.

## Figures and Tables

**Table 1:** Chemical composition of epoxy in carbon fiber-reinforced polymer (CFRP).

Ingredient	Concentration (%)
Bisphenol-A epoxy resin	37–38
Novolac epoxy resin	19–20
Dicyandiamide	5–6
Methyl ethyl ketone (MEK)	36–37

**Table 2:** Parameters and test results used for tensile tests after polarization.

Specimen	*A*_s_^2^ (mm^2^)	*A*_c_^3^ (mm^2^)	*i* ^4^ (A/m^2^)	*q* ^5^ (10^7^ C/m^2^)	*f*_u_^6^ (MPa)	Failure modes	*K*_Exp_^9^	*K*_cal_^10^/*K*_Exp_
I0-D25	653.00	25.24	0	0	727.74	L ^7^	1.07	—
I0-D25# ^1^	658.75	25.73	0	0	760.30	L	1.11	—
I0.5-D25	617.35	24.83	0.925	0.200	510.17	L	0.75	1.19
I0.5-D25#	592.43	26.72	0.963	0.208	601.65	L	0.88	1.00
I1-D25	590.40	25.72	1.837	0.397	564.80	L	0.83	0.95
I1-D25#	630.96	25.04	1.722	0.372	522.46	L	0.77	1.04
I2-D25	632.64	25.90	3.156	0.682	460.45	D ^8^	0.67	0.98
I2-D25#	630.48	26.27	3.164	0.683	521.01	D	0.76	0.86
I4-D25	618.76	25.94	6.456	1.394	413.11	D	0.60	0.71
I4-D25#	646.07	25.84	6.167	1.332	276.91	D	0.41	1.10
I0-D50	670.40	25.14	0	0	669.81	L	0.98	—
I0-D50#	667.59	25.80	0	0	573.48	L	0.84	—
I0.5-D50	629.28	25.63	0.924	0.399	532.11	L	0.78	1.01
I0.5-D50#	630.00	25.33	0.904	0.390	605.18	L	0.89	0.89
I1-D50	617.82	24.19	1.739	0.751	364.62	D	0.53	1.19
I1-D50#	643.62	25.21	1.700	0.734	435.73	D	0.64	1.00
I2-D50	645.09	22.91	3.129	1.352	243.59	D	0.36	1.23
I2-D50#	618.05	25.84	3.266	1.411	255.54	D	0.37	1.13
I4-D50	630.24	24.82	6.297	2.720	162.84	D	0.24	0.80
I4-D50#	612.17	25.54	6.487	2.803	67.50	D	0.10	1.84
Mean	—	—	—	—	—		—	1.06
COV	—	—	—	—	—		—	0.239

^1^ # = duplicate specimen, ^2^ *A*_s_ = measured anodic surface area, ^3^ *A*_c_ = measured cross-sectional area, ^4^ *i* = actual current densities, ^5^ *q* = charge density, ^6^ *f*_u_ = ultimate tensile strength, ^7^ L = lateral failure type, ^8^ D = edge delamination failure type, ^9^ *K*_Exp_ = experimental deterioration factor, ^10^ *K*_Cal_ = calculated deterioration factor.
